# Exploring the Impact of the *APOE3‐Christchurch* Mutation on APOE Biology Using iPSC‐derived Cortical Organoids

**DOI:** 10.1002/alz70855_107229

**Published:** 2025-12-25

**Authors:** Ana‐Caroline Raulin, Andrew Keaton Gjelsteen, Wenyan Lu, Paula Rodriguez Martinez, Jacob Marsh, Ailsa Crow, Yuka A Martens, Yasuteru Inoue, Hu Wang, Xianlin Han, Celeste M. Karch, Guojun Bu, Julia TCW, Takahisa Kanekiyo

**Affiliations:** ^1^ Mayo Clinic, Jacksonville, FL, USA; ^2^ Boston University, Boston, MA, USA; ^3^ Washington University in St Louis, St Louis, MO, USA; ^4^ UT Health San Antonio, San Antonio, TX, USA; ^5^ University of Texas Health Sciences Center, San Antonio, San Antonio, TX, USA; ^6^ Department of Psychiatry, Washington University in St. Louis School of Medicine, St. Louis, MO, USA; ^7^ Boston University Chobanian & Avedisian School of Medicine, Boston, MA, USA

## Abstract

**Background:**

The rare *APOE3*‐Christchurch (*APOE3Ch*) variant is associated with remarkable protective effects, delaying the age at onset of *PSEN1*‐E280A driven autosomal dominant Alzheimer's disease (AD). While much of the research on *APOE3C*h has focused on its role in AD, little is known about how this variant impacts APOE biology under non‐disease conditions. Using cortical organoids as a model system, we aim to investigate the fundamental mechanisms by which *APOE3Ch* alters APOE biology, including its effects on metabolism and cellular interactions, to better understand its unique properties and potential implications for broader applications.

**Method:**

Cortical organoids were derived from the isogenic neurodegeneration iPSC line series, with either the *APOE3* or *APOE3Ch* genotype. Single‐cell RNA sequencing (scRNA‐seq) was performed on three independent batches of 6‐month‐old cerebral organoids to identify pathways differentially regulated by APOE3Ch. To further investigate APOE lipidation and its effects on lipid metabolism, lipidomics analysis was conducted on 8‐month‐old cerebral organoids, offering additional insights into *APOE3Ch*‐specific biological mechanisms. Validation was also carried out using iPSC‐derived astrocytes and neurons to confirm key findings.

**Result:**

ScRNA‐seq revealed differences in cell populations and astrocyte subtypes between *APOE3* and *APOE3Ch* organoids, with mitochondrial‐related pathways differentially regulated in *APOE3Ch* cerebral organoids. These findings were validated in iPSC‐derived astrocytes. *APOE3Ch* iPSC‐derived astrocytes exhibited increased APOE secretion compared to *APOE3*. They also demonstrated enhanced mitochondrial function, as evidenced by increased spare respiratory capacity measured by Seahorse stress assays. Furthermore, *APOE3Ch* astrocytes showed reduced lipid droplet formation following oleic acid treatment. Lipidomics analysis of *APOE3Ch* cerebral organoids revealed decreased cholesterol ester levels, suggesting alterations in lipid metabolism.

**Conclusion:**

Our findings provide novel insights into how the *APOE3Ch* variant alters APOE biology, revealing differences in mitochondrial function and lipid metabolism. The differential regulation of mitochondrial pathways in *APOE3Ch* cerebral organoids, along with the enhanced mitochondrial capacity and altered lipid metabolism observed in *APOE3Ch* astrocytes, highlights the unique properties of this variant. These results offer valuable mechanistic understanding that may inform future therapeutic strategies targeting APOE biology, particularly in the context of AD.

